# Chloroform: MAK Value Documentation, addendum – Translation of the German version from 2022

**DOI:** 10.34865/mb6766e10_1ad

**Published:** 2025-03-31

**Authors:** Andrea Hartwig, MAK Commission

**Affiliations:** 1 Institute of Applied Biosciences. Department of Food Chemistry and Toxicology. Karlsruhe Institute of Technology (KIT) Adenauerring 20a, Building 50.41 76131 Karlsruhe Germany; 2 Permanent Senate Commission for the Investigation of Health Hazards of Chemical Compounds in the Work Area. Deutsche Forschungsgemeinschaft, Kennedyallee 40, 53175 Bonn, Germany. Further information: Permanent Senate Commission for the Investigation of Health Hazards of Chemical Compounds in the Work Area | DFG

**Keywords:** chloroform, nose, liver, kidney, carcinogenicity, MAK value, maximum workplace concentration, peak limitation, developmental toxicity, skin absorption

## Abstract

The German Commission for the Investigation of Health Hazards of Chemical Compounds in the Work Area has re-evaluated the occupational exposure limit value (maximum concentration at the workplace, MAK value) of chloroform [67-66-3] considering all toxicological end points. Relevant studies were identified from a literature search and also unpublished study reports were used. There are no human data to derive a MAK value. In 1999, a NOAEC of 5 ml/m^3^ was determined in a 13-week inhalation study based on the increased cell proliferation in the kidneys and liver of rats and mice. A MAK value of 0.5 ml/m^3^ was established on the basis of these findings. This value has now been confirmed also after taking the increased respiratory volume at the workplace into account (see List of MAK and BAT values, Section I b and I c). In a new 2-year inhalation study, respiratory metaplasia of the olfactory epithelium and thickening of the bone in the nasal cavity was observed at the lowest chloroform concentrations tested of 10 ml/m^3^ in rats and 5 ml/m^3^ in mice. Toxicity in the olfactory epithelium can be a local as well as a systemic effect. After considering the two possible modes of action and the increased respiratory volume at the workplace, the MAK value derived from effects in the nose would correspond to 1 ml/m^3^. The present MAK value of 0.5 ml/m^3^ thus protects also against effects in the nose. As the critical effect is systemic, chloroform remains classified in Peak Limitation Category II with an excursion factor of 2. A developmental toxicity study with inhalation exposure of rats determined a NOAEC of 10 ml/m^3^. Additionally, malformations in rats observed in another developmental toxicity study at 100 ml/m^3^ are suggestive of teratogenicity. The NOAEC in this study was 30 ml/m^3^. Furthermore, the NOAEL for developmental toxicity after gavage administration was 50 mg/kg body weight and day in rats and 35 mg/kg body weight and day in rabbits. Overall, the margins between the concentrations at the workplace calculated to be without effects and the MAK value are sufficient. Therefore, as damage to the embryo or foetus is unlikely to occur if the MAK value is not exceeded, chloro­form remains assigned to Pregnancy Risk Group C. Chloroform causes tumours of the thyroid gland in rats and liver tumours in mice as well as renal tumours in both species. However, chloroform is not expected to induce these tumours via a genotoxic mode of action and has thus been classified in Carcinogen Category 4. There are no data for sensitizing effects in humans and no reliable positive results from animal and in vitro studies. Data for sensitization of the respiratory tract are not available. Uptake via the skin can contribute significantly to systemic toxicity. Therefore, chloroform remains designated with an “H”.

**Table:** 

**MAK value (1999)**	**0.5 ml/m^3^ (ppm) ≙ 2.5 mg/m^3^**
**Peak limitation (2001)**	**Category II, excursion factor 2**
	
**Absorption through the skin (1999)**	**H**
**Sensitization**	**–**
**Carcinogenicity (1999)**	**Category 4**
**Prenatal toxicity (1999)**	**Pregnancy Risk Group C**
**Germ cell mutagenicity**	**–**
	
**BAT value**	**–**
	
Synonyms	methyl trichloride trichloromethane
Chemical name (IUPAC)	chloroform
CAS number	67-66-3
Structural formula	HCCl_3_
Molecular formula	CHCl_3_
Molar mass	119.38 g/mol (ECHA 2020)
Melting point	–63.5 °C (ECHA 2020)
Boiling point	61 °C (ECHA 2020)
Density at 20 °C	1.49 g/cm^3^ (ECHA 2020)
Vapour pressure 20 °C	211 hPa (ECHA 2020)
log K_OW_	1.97 (ECHA 2020)
Solubility	8700 mg/l water (ECHA 2020)
**1 ml/m^3^ (ppm) ≙ 4.954 mg/m^3^**	**1 mg/m^3^ ≙ 0.202 ml/m^3^ (ppm)**

Documentation for chloroform was published in 1977 followed by an addendum on prenatal toxicity in 1989, an addendum in 1999 (Greim 2000, combined in one translation), an addendum on peak limitation in 2001 (Greim 2001, available in German only) and an addendum on genotoxicity in 2003 (Hartwig 2010).

In 2016, the Commission began using a revised approach for assessing substances with a MAK value based on systemic effects and derived from inhalation studies in animals or studies with volunteers at rest; this new approach takes into account that the respiratory volume at the workplace is higher than under experimental conditions. However, this does not apply to gases or vapour with a blood:air partition coefficient < 5 (see List of MAK and BAT Values, Section I b and I c). The blood:air partition coefficient for chloroform is 7.43 (Corley et al. 1990). This addendum evaluates whether the MAK value and the pregnancy risk group need to be revised as a result of the higher respiratory volume at the workplace. The addendum reviews also recently published findings that are relevant to the individual end points. Cited unpublished toxicological studies from companies have been made available to the Commission.

Chloroform was used as an inhalation anaesthetic until the end of the 19^th^ century. For a time, it was the most commonly applied anaesthetic in Europe. Today, it is used as a solvent and for chemical synthesis. The main application of chloroform was its conversion to chlorodifluoromethane, a precursor to polytetrafluoroethylene. However, the import and use of chlorodifluoromethane has been prohibited in Germany since 2000 and in Europe since 2015 (EU 2009). Additionally, chloroform is a constituent of cleaning agents and disinfectants or forms in these agents as a by-product. For example, chloroform is produced during the treatment of drinking water with sodium hypochlorite or chlorine dioxide (IARC 1999).

## 1 Toxic Effects and Mode of Action

In humans, not only neurological effects, but also chronic kidney and liver damage as well as acute shortness of breath and arrhythmia were induced at high concentrations of chloroform such as previously used for anaesthesia (12 000–48 000 mg/m^3^) (Greim 2000).

After oral and inhalation exposure of rats and mice to chloroform, the most sensitive target organs are the nose and the kidneys as well as the liver. Exposure to chloroform causes tumours in the thyroid gland and kidneys in rats and tumours in the kidneys and liver of mice. There is no evidence of a genotoxic mechanism of action. There are still no reliable findings of a sensitization potential for chloroform. In developmental toxicity studies, inhalation exposure of rats to chloroform induced developmental delays in the form of reduced body weights and decreased crown–rump lengths concurrently with maternal toxicity at concentrations of 30 ml/m^3^ and above. Signs of teratogenicity were found at concentrations of 100 ml/m^3^ and above. Chloroform given by gavage induced developmental delays in rats at doses of 126 mg/kg body weight and day and above and in rabbits at a dose of 50 mg/kg body weight and day; these effects were observed concurrently with maternal toxicity. Effects on fertility occurred only at a chloroform dose of about 1000 mg/kg body weight and day and concurrently with maternal toxicity.

## 2 Mechanism of Action

### 2.1 Nephrotoxicity

As described in the addendum from 1999 and in Section 3.2, reactive intermediate products such as phosgene and the dichloromethyl radical form during the metabolism of chloroform. These products can react with cellular components. However, a genotoxic mechanism of action is not assumed for chloroform. Tumours in the liver and kidneys were observed only concurrently with toxicity. For this reason, regenerative hyperplasia is considered decisive for the carcinogenic effects of chloroform (Greim 2000).

A study investigated the mechanism of action of chloroform-associated renal toxicity in male mice that do not express NADPH-cytochrome P450 oxidoreductase (CPR) in the liver. As a result, the activity levels of cytochrome P450 (CYP) are about 95% lower in the liver microsomes of these mice. In comparison with the levels found in wild-type mice, the liver-CPR-null mice had higher chloroform concentrations in the blood, kidneys and liver and increased renal toxicity at a dose of 150 mg/kg body weight. According to the authors, the increase in renal toxicity was caused by the greater bioavailability of chloroform in the blood. The authors suggested that CYP2E1 in the kidneys plays a critical role in the induction of renal toxicity by chloroform and that the metabolic activation of chloroform in the liver plays a subordinate role. According to the authors, the highly reactive metabolite phosgene reacts rapidly with cellular proteins in the liver, making it unlikely that phosgene is transported from the liver to other organs such as the kidneys (Fang et al. 2008).

### 2.2 Effects on the nose

The mechanism of action underlying the toxicity of chloroform in the nose of female B6C3F1 mice and male F344 rats was investigated after exposure to chloroform concentrations of 1, 3, 10, 30, 100 and 300 ml/m^3^ for 6 hours a day on 7 days. In female mice, new bone formation in the proximal region of the endoturbinate I (dorsal nasal concha) was induced at concentrations of 300 ml/m^3^ and above. At chloroform concentrations of 10 ml/m^3^ and above, cell proliferation was increased in this region with statistical significance in comparison with the values found in the controls. In male rats, the effects were found mainly in two regions of the nasal cavity. At concentrations of 10 ml/m^3^ and above, goblet cells in the nasal passage were enlarged and diverse changes in the ethmoid region (lamina propria of the olfactory mucosa and the underlying bones) were observed including new bone formation in the nasal concha, atrophy of the Bowman’s glands and increased cell proliferation. The effects were more severe in the lower region of the Bowman’s glands and in the overlaying olfactory epithelium and did not occur in the upper region of the Bowman’s glands. The highest increase in cell proliferation was observed in the proximal and central regions of the endoturbinate I, while only a moderate increase was detected in the distal region. In the rats of the control group, immunohistochemical staining revealed CYP2E1 mainly in the cytoplasm of the olfactory epithelium and in the upper region of the Bowman’s glands. No damage induced by chloroform was observed in these tissue layers. On the basis of these findings, the authors concluded that CYP2E1 does not play a critical role in the development of olfactory toxicity in the nose. The effects induced by chloroform in the olfactory mucosa are evident in the lower layers of the lamina propria and thus not in the main respiratory passage of the rat. According to the authors, the location of the toxic effects suggests that the adverse effects in the nasal mucosa are not caused by the deposition of chloroform after inhalation, but by the distribution of chloroform or its toxic metabolites via the blood. About 0.5% of the cardiac output reaches the respiratory tract of the rat via the circulation of the blood (Méry et al. 1994). The effects described suggest that the toxicity in the nose induced by chloroform after inhalation are caused by the systemic distribution of the substance and not by local effects. This was confirmed by the effects induced by chloroform in the nose after oral administration. After chloroform was given to F344 rats by gavage for 4 days or 3 weeks, effects such as new bone formation and increased cell replication in the olfactory mucosa lining of the ethmoid region were observed at doses of 34 mg/kg body weight and day and above. The effects in the liver and kidneys first became noticeable at chloroform doses of 100 mg/kg body weight and day and above (Larson et al. 1995). The pathomechanism of bone thickening can be explained by a degeneration of the Bowman’s glands that leads to irritation of the periosteum and subsequent proliferation of bone cells.

A study with naphthalene was used to compare chloroform with another substance that induces local and systemic effects in the nose. This study investigated the effects induced in Sprague Dawley rats after exposure to naphthalene by inhalation and intraperitoneal injection. Cellular damage was found only in the olfactory mucosa of the nose after exposure via both routes of administration, suggesting that damage occurs in the olfactory epithelium only after metabolic conversion. After inhalation exposure to naphthalene, the damage was confined to the medial meatus, the main passage for air flow through the nose. Regions with less air flow, such as the ethmoturbinates, were not affected by exposure via the inhalation route. By contrast, damage was found also in the regions of the ethmoturbinates after systemic administration by intraperitoneal injection (Lee et al. 2005). Therefore, the pattern of damage to the nose found in the studies of Lee et al. (2005) and Méry et al. (1994) suggests that chloroform does not induce effects in the main respiratory passage of the nose of rats, which indicates a systemic effect.

However, there are also arguments in favour of a local mechanism of action for the toxicity in the nose. The toxico­kinetics data after inhalation exposure of rodents and humans (see Section 2.1) show that unchanged chloroform passes through the respiratory tract not only after inhalation, but also after exhalation. It is assumed that chloroform is converted into toxic metabolites by the metabolic enzymes in the nose during inhalation and exhalation, leading to damage in the nasal epithelium. A local mechanism of action is suggested also by the finding that the effects induced in rats and mice after inhalation of chloroform, such as degeneration of the olfactory epithelium (see Section 5.2.1), are consistent with the effect pattern of local irritation.

**Summary**: The toxic effects induced in the nose after inhalation of chloroform may be caused by both systemic (after distribution via the bloodstream) and local (via direct effects at the point of contact) mechanisms of action.

## 3 Toxicokinetics and Metabolism

### 3.1 Absorption, distribution, elimination

The data for toxicokinetics were already included in the documentation published in 1977 and in the addendum from 1999 (Greim 2000).

B6C3F1 mice and Osborne Mendel rats were exposed for 6 hours by inhalation to [^14^C]-labelled chloroform in concentrations of 10, 89 or 366 ml/m^3^ and 93, 356 or 1041 ml/m^3^, respectively. Of the total radioactivity absorbed (as sum of exhaled chloroform, CO_2_, in the urine, faeces and in the carcass), mice exhaled 0.3%, 0.5% and 8% and rats 2%, 20% and 42% as chloroform within 48 hours after the end of exposure. It was not possible to determine the amount exhaled during exposure. Most of the radioactivity was exhaled as CO_2_; in mice, this occurred already during exposure. At higher concentrations, metabolism to CO_2_ was limited (Corley et al. 1990; Greim 2000).

The blood:air partition coefficients for chloroform in rats, mice and humans are 21.3, 20.8 and 7.43, respectively (Corley et al. 1990). Therefore, at the same level of external exposure, rats and mice absorb more of the substance into the blood than humans.

In animals, chloroform is absorbed rapidly and completely via the gastrointestinal tract. The radioactivity in the exhaled air, the faeces and the carcass was examined in mice and rats up to 48 hours after oral administration. In mice and rats, [^14^C]-CO_2_ accounted for the largest fraction determined in the exhaled air (85% and 67%, respectively), followed by unchanged chloroform (6% and 20%, respectively). In comparison, monkeys exhaled 79% in unchanged form and 18% as CO_2_. A total of 2% to 10% was excreted with the urine and faeces (WHO 1994). Within 8 hours after oral administration of 500 mg ^13^C-chloroform to test persons, 50% was recovered in the exhaled air as CO_2_ and up to 68% in unchanged form. After 1.5 hours, the maximum concentrations of chloroform in the blood were 1 to 5 mg/l (Fry et al. 1972). After inhalation of a single dose of 5 mg [^38^Cl]-labelled chloroform (equivalent to 200 ml/m^3^) as a vapour with a subsequent breath-hold for 20 seconds, test persons retained 80%. Within an hour after exposure, 10% of the absorbed radioactivity had been exhaled (Greim 2000; Morgan et al. 1970). Humans have been found to absorb chloroform through the skin (Borzelleca and Carchman 1982; Greim 2000).

Twelve male and female test persons between 18 and 50 years of age were orally administered a gelatine capsule containing 500 mg chloroform and 1 ml olive oil. The half-lives in the blood of 4 test persons were determined. The authors observed biphasic elimination with initial half-lives of 9, 21, 12 and 14 minutes and terminal half-lives of 96, 92, 86 and 86 minutes (no other details) (Fry et al. 1972). However, a discrepancy was found between the diagram illustrating the concentration–time course in the blood and the values given for the initial half-life.

### 3.2 Metabolism

**Fig.1:**
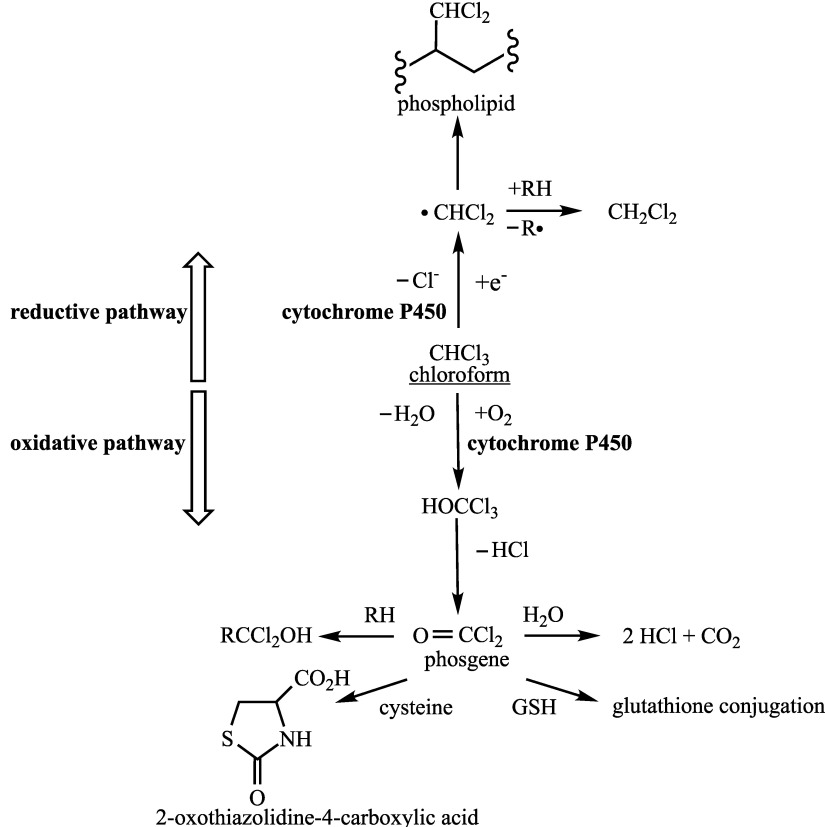
Diagram of the metabolic pathway of chloroform (according to WHO 1994)

The metabolism of chloroform (Figure 1) has been studied extensively and was described in the addendum from 1999 (Greim 2000). Chloroform undergoes metabolism in the liver via both an oxidative and a reductive pathway depending on the substrate concentration and the amount of oxygen present in the cell. In humans, the primary pathway is the oxidative metabolism by CYP to form trichloromethanol, which is converted by dehydrochlorination to phosgene. Phosgene reacts with water to yield HCl and CO_2_ or forms unstable adducts with biological macromolecules. Additionally, phosgene is able to react directly with cysteine to form 2-oxothiazolidine-4-carboxylic acid or is detoxified via glutathione (GSH). However, the metabolites may react with proteins if the GSH status is reduced.

Under reductive conditions, chloroform is metabolized to the dichloromethyl radical, also via CYP. This radical, in turn, is either converted to dichloromethane or reacts with fatty acids and phospholipids. The reductive metabolic pathway, however, plays a subordinate role in the human liver because it takes place only at high substrate concentrations and under anaerobic conditions. Oxidative metabolism is the primary pathway under in vivo conditions (Borzelleca and Carchman 1982).

CYP2E1 plays a decisive role in the formation of toxic metabolites and thus in the toxicity of chloroform in the liver, kidneys and nose. Mainly smaller planar molecules are metabolized by CYP2E1. In rat liver microsomes, oxidation to phosgene was catalysed at low chloroform concentrations by CYP2E1 and at higher concentrations of 5 mM primarily by the isoenzymes CYP2B1/2 and CYP2C11 (Docks and Krishna 1976; Gemma et al. 2003; Greim 2000; Zanger and Schwab 2013). Evidence of CYP2E1 was found by Western and Northern blot analysis in the protein and RNA in the liver, the kidneys and the nasal mucosa of Sprague Dawley rats given gavage doses of acetone of 5 ml/kg body weight twice a day on 2 consecutive days. After 24 hours, CYP2E1 protein levels and CYP2E1 enzyme activity were increased in all 3 tissues in comparison with the levels determined in the control rats (detected by hydroxylation of 4-nitro­phenol). An additional statistically significant increase in CYP2E1-mRNA was found only in the nasal mucosa (Longo and Ingelman-Sundberg 1993). In human liver microsomes, phosgene formed from chloroform mainly via the action of the enzyme CYP2A6 under oxidative conditions and the saturation of CYP2E1 (Gemma et al. 2003; Guengerich et al. 1991). The protein of CYP2E1 was not detected in human kidney microsomes (Amet et al. 1997; Cummings et al. 2000).

Nephrotoxicity was more pronounced in male than in female mice after administration of chloroform because of higher CYP levels in the kidneys (Smith et al. 1984). Immunoblotting confirmed that CYP isoenzymes such as CYP2B1, CYP2C6, CYP2E1 or CYP4A1 are expressed more strongly in male C57Bl/6 and DBA/2N mice than in the female mice of these strains (Wolf 1991). This difference was observed also by the studies included in this addendum (Section 5). However, it was not found in rats, rabbits or guinea pigs and thus seems to be species-specific (ATSDR 1997). In general, CYP2E1 regulation is complex and its expression is influenced by multiple factors such as xenobiotics, hormones (particularly growth hormones, testosterone, thyroid hormones and insulin) as well as diseases such as inflammation, obesity and diabetes (Overton et al. 2008). Testosterone may also play a noteworthy role; this is supported by findings of an increase in renal toxicity after female ICR mice treated with testosterone were given chloroform by subcutaneous injection and the absence of renal toxicity in castrated male mice (Smith et al. 1984).

#### 3.2.1 Differences in the nasal tissue of rodents and humans

Studies of metabolism in nasal tissue were carried out mainly in rodents. There are limited data for the nasal mucosa in humans because of the difficulties in taking samples of human nasal tissue.

The differences in nasal metabolism in rodents and humans are influenced by both anatomy and physiology, but are mainly due to the differing enzymes in the nose. Unlike humans, rats and mice breathe exclusively through the nose and, as described in Section 3.1, absorb more chloroform into the blood than humans at the same level of external exposure (Corley et al. 1990; Morris et al. 2010). Overall, the nose of the rodent is therefore exposed to higher concentrations of chloroform.

Another difference between the rodent and human nose is that about 15% of the inhaled air flows over the olfactory epithelium in rats, while only 7% of the air flows along this path in humans (Harkema et al. 2006; Morris et al. 2010).

The olfactory epithelium covers about 50% and 47% of the nasal cavity in rats and mice, respectively, but only 3% of that in humans. The Bowman’s glands occur throughout the lamina propria of the olfactory epithelium. As rats have a much higher percentage of olfactory epithelium than humans, also the fraction of Bowman’s glands in rats is noticeably larger than that in humans. Therefore, substances that cause effects in these nasal tissues have a much greater potency in rats (Gross et al. 1982; Harkema and Morgan 1996).

Unlike in humans, also the respiratory epithelium in rodents has metabolic potential because it contains a non-ciliated transitional epithelium with higher metabolic activity. For this reason, the respiratory epithelium in humans is considered to have lower metabolic competence than that in rats (Morris et al. 2010).

The olfactory epithelium contains a much higher level of metabolizing enzymes than the respiratory epithelium. This was observed in various species including cynomolgus monkeys (Macaca fascicularis), dogs and rats (Kassinova et al. 1981; Mehta and von Borstel 1982; Scheel and Zimmermann 1981). Not only the protein levels, but also the catalytic activity of the CYP enzymes is increased in the olfactory epithelium. The catalytic activity of the CYP enzymes in the nasal epithelium is even higher than the activity levels found in other tissues including those of the liver (Sarkar 1992). This is assumed to be associated with NADPH reductase levels, the enzyme responsible for electron transfer in the CYP reaction cycle and thus for the rate of the reaction. A study with rats, mice and hamsters found that the ratio of NADPH reductase to CYP in all species was 1:2 to 1:3 in the olfactory epithelium compared with a ratio of 1:11 to 1:15 in the liver (Reed et al. 1986). In male F344 rats, the enzyme CYP2E1 that is relevant for chloroform toxicity was found primarily in the Bowman’s glands, the supporting cells of the olfactory epithelium, in the ciliated cells of the respiratory epithelium and in the non-secretory cells of the transitional epithelium (Reed 1993). To date, no studies have detected the expression of CYP2E1 in proteins from human nasal tissue. On a transcriptional level, *CYP2E1 *was identified in the nasal mucosa of human foetuses by qualitative polymerase chain reaction (Zhang et al. 2005). A number of studies detected the expression of the genes *CYP2A6, 2A13, 2C* and *3A*, but not of *CYP2E1*, in the nasal mucosa of adult humans (Ding and Kaminsky 2003). *CYP2E1* transcription and protein levels were only slightly correlated (Zanger and Schwab 2013). This is explained by the short half-life of CYP2E1 and its stabilization by the substrate. In the presence of substrate, the half-life is longer and proteasomal degradation does not take place.

An earlier study determined only slight CYP2E1 enzyme activity based on the de-ethylation of diethylnitrosamine in the human nasal mucosa (Longo et al. 1989). In a more recent study, no CYP2E1 enzyme activity was detected in the human nasal tissue. This study used microsomes from the respiratory and olfactory tissues of rats, mice and humans to analyse the rate of metabolism of styrene to styrene oxide catalysed by CYP2E1 and CYP2F2. In rats and mice, metab­­olism to styrene oxide occurred at a similar rate in both tissues; however, the rate in the olfactory epithelium was about double that in the respiratory epithelium. Another finding in rats and mice was that the metabolic conversion to styrene oxide was higher in the respiratory and olfactory epithelium than in the liver. The metabolism of styrene to styrene oxide was not found to occur in human nasal tissue (Green et al. 2001).

#### 3.2.2 Summary

In comparison with rodents, humans are assumed to be less susceptible to the metabolic activation of chloroform to form toxic metabolites in the nose. This is explained by differences in the anatomy and physiology of the rodent nose; volatile substances are taken up by rodents via the nose in larger amounts, leading to greater contact between the substances and the metabolically active region, the olfactory epithelium. Furthermore, the olfactory epithelium makes up a larger fraction of the nasal epithelium in rats than in humans. The assumption that metabolism occurs at a far higher rate in the rat nose is further supported by the higher levels of CYP enzyme activity, also of CYP2E1, the enzyme relevant to the metabolic conversion of chloroform to phosgene. In rodents, CYP2E1 activity levels are assumed to be even higher in the nose than in the liver. As more Bowman’s glands occur in rats than in humans, rats are regarded as more susceptible to effects in this tissue.

## 4 Effects in Humans

### 4.1 Single exposures

There are no new data available.

### 4.2 Repeated exposure

Studies that investigated this end point are described in the documentation from 1977 and in the addendum from 1999 (Greim 2000). Additionally, the report of the US EPA (2001) includes an extensive review of the observational data after repeated exposure of humans to chloroform. Studies following exposure to chloroform in drinking water are described, however, co-exposure to other trihalomethanes and additional disinfectants also occurred. The report includes also workplace studies that do not specify the chloroform concentrations and that involved concurrent exposure to other chemicals (US EPA 2001). For this reason, the studies are not suitable for the evaluation of the effects after repeated exposure.

### 4.3 Local effects on skin and mucous membranes

There are no new data available.

### 4.4 Allergenic effects

There are no new data available.

### 4.5 Reproductive and developmental toxicity

In a review article (Williams et al. 2018), 30 studies on the association between reproductive toxicity and chloroform exposure were identified following extensive research. In 29 of the studies, exposure levels were determined by self-reported behaviour with respect to showering, bathing, hand washing, the use of a water filter, tap water use or based on regional data for chloroform concentrations in the drinking water. One of the studies reported a median chloroform concentration in the blood of 50.7 ng/l. In about half of the 30 studies, no association was found between exposure to chloroform and birth weight. However, a definite conclusion cannot be drawn because the data for exposure were inadequate. In 13 of 17 studies that investigated the parameter “too small for gestational age”, no statistically significant association between exposure to chloroform and foetal growth was found. The other 4 studies found that the increase in the risk of “too small for gestational age” was marginally statistically significant at high levels of exposure to chloroform. The authors reported that a causal relationship between exposure to chloroform and an effect on foetal growth cannot be derived because of the inconsistent findings and a lack of significant findings in studies that reported higher quality data for the analysed levels of chloroform exposure. In addition, exposure to chloroform was not associated with premature births.

A study reported evidence of a possible association between exposure to chloroform and defects in the cardiac septum or neural tube. However, the findings do not show a clear dose–response relationship and are not supported by the findings from other epidemiological studies (Williams et al. 2018). As the studies do not provide quantitative data for exposure to chloroform, they are not included in the evaluation of reproductive toxicity.

### 4.6 Genotoxicity

There are no new data available.

### 4.7 Carcinogenicity

There are no new data available.

## 5 Animal Experiments and in vitro Studies

### 5.1 Acute toxicity

#### 5.1.1 Inhalation

Effects induced after a single exposure by inhalation are described in the addendum from 1999 (Greim 2000). A 4-hour LC_50_ value of 10 500 mg/m^3^ for rats is reported (ECHA 2020).

#### 5.1.2 Oral administration

The oral LD_50_ values were in a range from 36 to 1366 mg/kg body weight for various mouse strains and in a range from 894 to 2000 mg/kg body weight for Sprague Dawley rats. Symptoms of toxicity were fatigue, reduced muscle tone, ataxia, piloerection and prostration (Greim 2000).

#### 5.1.3 Dermal application

Two weeks after occlusive application of a single chloroform dose of 1000 or 4000 mg/kg body weight for 24 hours, severe, dose-dependent degenerative changes in the renal tubules were observed in 2 rabbits. There were no effects in the liver (Torkelson et al. 1976).

### 5.2 Subacute, subchronic and chronic toxicity

#### 5.2.1 Inhalation

Studies that investigated the chronic toxicity of chloroform were discussed in detail in the addendum from 1999 (Greim 2000). Chloroform causes toxic effects in the liver and kidneys. It was previously unclear how the minimal effects on the olfactory epithelium of the nose are to be evaluated. In the following, inhalation studies that are rele­vant for the evaluation are presented and discussed again together with findings that were published after 1999. Additionally, new data are available for the effects induced by chloroform in the nasal cavity.

##### 5.2.1.1 2-Week study

Groups of 10 male and 10 female F344 rats and groups of 10 male and 10 female BDF1 mice were exposed to chloroform concentrations of 0, 500, 1000, 2000, 4000 or 8000 ml/m^3^ for 6 hours a day, on 5 days a week, for 2 weeks. All male and female mice and rats in the groups exposed to chloroform concentrations of 2000 ml/m^3^ and above died. In the male mice that survived, necrosis and cytoplasmic basophilia of the proximal tubules of the kidneys and slight swelling and vacuolization of the liver were observed at chloroform concentrations of 500 and 1000 ml/m^3^. Atrophy and respiratory metaplasia were detected in the olfactory epithelium. In the female mice, necrosis and vacuolization in the central region of the liver as well as degeneration, necrosis and restructuring in the olfactory and respiratory epithelium of the nose were observed at these concentrations. In the surviving female and male rats of the 500 ml/m^3^ and 1000 ml/m^3^ groups, vacuolization was noticeable in the proximal tubules of the kidneys and in the central region of the liver. In the nasal cavity, desquamation, atrophy and restructuring in the olfactory epithelium as well as oedema in the lamina propria were observed (Kasai et al. 2002).

##### 5.2.1.2 13-Week studies

Groups of 10 male and 10 female F344 rats and groups of 10 male and 10 female BDF1 mice were exposed in an exposure chamber to chloroform concentrations of 0, 12, 25, 50, 100 or 200 ml/m^3^ for 6 hours a day, on 5 days a week, for 13 weeks. High mortality was observed among male mice shortly after the beginning of exposure (see Table 1); this was probably caused by necrosis of the proximal renal tubules. All female mice and all rats survived the 13-week exposure period. The histopathological findings in the liver, kidneys and nasal cavity are shown in Table 2 and Table 3.

**Tab.1:** Mortality of BDF1 mice after 13-week exposure to chloroform (Kasai et al. 2002)

	♂	♀
Number of animals	10	10
**Concentration [ml/m^3^]**		
0	0	0
12	2 (1/1^st^)(1/3^rd^)	0
25	9 (8/1^st^)(1/3^rd^)	0
50	10 (7/1^st^)(3/2^nd^)	0
100	8 (8/1^st^)	0
200	10 (10/1^st^)	0

1^st^, 2^nd^, 3^rd^: day of exposure

**Tab.2:** Histopathological findings in BDF1 mice after 13-week exposure to chloroform (Kasai et al. 2002)

	Concentration [ml/m^3^]					
		0	12	25	50	100	200
Number of animals	♂	10	10	10	10	10	10
	♀	10	10	10	10	10	10
**nasal cavity**							
degeneration of the olfactory epithelium	♂	0	2	5**	7**	6**	9**
	♀	0	0	0	0	0	0
bone thickening	♂	0	7**	1	0	1	0
	♀	0	7**	10**	9**	9**	9**
eosinophilic changes in the olfactory epithelium	♂	0	0	0	0	1	0
	♀	0	7**	9**	8**	9**	10**
eosinophilic changes in the respiratory epithelium	♂	0	0	0	0	1	0
	♀	0	8**	8**	7**	7**	8**
**liver**							
swelling	♂	0	0	0	0	0	10**
	♀	0	0	0	0	0	0
cell atypia	♂	0	0	0	0	0	0
	♀	0	0	0	0	10**	10**
necrosis	♂	0	0	0	0	0	0
	♀	0	0	0	0	0	10**
vacuolization	♂	0	0	0	0	0	0
	♀	0	0	0	0	0	2
**kidneys (proximal tubules)**							
tubular necrosis	♂	0	7**	10**	10**	9**	10**
	♀	0	0	0	0	0	0
degeneration	♂	0	2	9**	10**	8**	10
	♀	0	0	0	0	0	0
cytoplasmic basophilia	♂	0	8**	1	0	2	0
	♀	0	0	0	0	1	0

**p ≤ 0.01 according to the chi-squared test

**Tab.3:** Histopathological findings in F344 rats after 13-week exposure to chloroform (Kasai et al. 2002)

		Concentration [ml/m^3^]					
		0	25	50	100	200	400
Number of animals	♂	10	10	10	10	10	10
	♀	10	10	10	10	10	10
**nasal cavity**							
mineralization	♂	0	10**	10**	10**	10**	10**
	♀	0	10**	10**	10**	10**	10**
atrophy of the olfactory epithelium	♂	0	7**	10**	10**	9**	10**
	♀	0	10**	10**	10**	10**	10**
necrosis of the olfactory epithelium	♂	0	0	0	0	8**	10**
	♀	0	0	0	0	0	1
**liver (central)**							
deposit of ceroid	♂	0	0	0	0	0	10**
	♀	0	0	0	0	8**	10**
structural collapse	♂	0	0	0	0	10**	10**
	♀	0	0	0	8**	9**	9**
vacuolization	♂	0	0	0	0	1	0
	♀	0	0	0	0	2	0
**kidneys (proximal tubules)**							
vacuolization	♂	0	0	0	0	3	4*
	♀	0	0	0	2	6**	5**

*p ≤ 0.05

**p ≤ 0.01 according to the chi-squared test

In female **mice**, bone thickening and eosinophilic changes in the olfactory and respiratory epithelium were observed in the nasal cavity at chloroform concentrations of 12 ml/m^3^ and above. In male mice, bone thickening was observed in the nasal cavity at a concentration of 12 ml/m^3^; however, a concentration–effect relationship was not evident. In the liver, swelling was observed in the male animals at 200 ml/m^3^ and above and cell atypia was found in the female animals at 100 ml/m^3^ and above. In the kidneys, changes were observed only in the male animals. Tubular necrosis and cytoplasmic basophilia were detected at chloroform concentrations of 12 ml/m^3^ and above. Significant degeneration of the proximal tubules was observed only in male mice at concentrations of 25 ml/m^3^ and above. In male and female **rats**, altered mineralization and atrophy of the olfactory epithelium were the most sensitive effects found at chloroform concentrations of 25 ml/m^3^ and above. Necrosis of the olfactory epithelium was detected only in male rats at concentrations of 200 ml/m^3^ and above. Structural deterioration of the liver was found in male rats at 200 ml/m^3^ and above and in female rats at 100 ml/m^3^ and above. Deposit of ceroid was observed in male rats at chloroform concentrations of 400 ml/m^3^ and above and in female rats at 200 ml/m^3^ and above. Significant changes in the vacuolization of the kidneys were observed in male animals at chloroform concentrations of 400 ml/m^3^ and above and in female animals at concentrations of 200 ml/m^3^ and above (Kasai et al. 2002). Overall, the most sensitive organ in **rats** was the **nose** with a **LOAEC** (lowest observed adverse effect concentration) of 25 ml/m^3^. Effects likewise developed in the **nose** and **kidneys** of **mice** at the **LOAEC** of **12 ml/m^3^**. A NOAEC (no observed adverse effect concentration) cannot be established. A particularly striking finding in the nose of mice was the differences in the effect patterns between the sexes. For example, degeneration in the olfactory epithelium was found only in male mice, while bone thickening and eosinophilic changes in the olfactory and respiratory epithelium were observed almost exclusively in female mice. This may be explained by the high mortality found among male mice shortly after the beginning of exposure to chloroform. An effect such as bone thickening would not have been noticeable yet because these kinds of effects are detected only in animals with longer lifespans. On the other hand, although it is possible that the olfactory epithelium in female mice may have undergone degeneration at an earlier time point of exposure, the olfactory epithelium may have regenerated by the end of the exposure period. The eosinophilic changes suggest that this form of regeneration may have occurred. The mortality in male mice, which the authors attribute to nephrotoxicity, agrees with an increased sensitivity of male mice to chloroform, leading to nephrotoxicity (see Section 3.2) (ATSDR 1997).

In another inhalation study, groups of 8 male and 8 female BDF1 mice were exposed to chloroform concentrations of 0, 5, 30 or 90 ml/m^3^ for 6 hours a day, on 5 days a week, for 13 weeks. Male mice were exposed additionally to a chloroform concentration of 1 ml/m^3^. The nose was not investigated in this study (Greim 2000; Templin et al. 1998). A concentration of **5 ml chloroform/m^3^** was established as the **NOAEC** for effects on the **liver** and **kidneys** of **mice** based on centrilobular swelling in the liver as well as a significant increase in cell proliferation and tubular regeneration in the renal cortex of male animals at a chloroform concentration of 30 ml/m^3^.

Groups of 5 to 15 female B6C3F1 mice were exposed to chloroform concentrations of 0.3, 2, 10, 30 or 90 ml/m^3^ for 6 hours a day, on 5 or 7 days a week, for exposure periods lasting 4 days or 3, 6 or 13 weeks. The findings of the 13-week study are shown in Table 9 (Greim 2000; Larson et al. 1996). From the findings of this study, a **NOAEC** of **2 ml/m^3^** was established for effects induced by **chloroform** in the **liver** of **mice** based on the increasing degree of swelling and vacuolization of the centrilobular hepatocytes and nephropathy (enlarged nuclei in the proximal tubule cells and a significant increase in cell proliferation in the kidneys) observed in mice at 10 ml/m^3^. There were no effects in the nose. These findings are in contrast to those of the 13-week study in BDF1 mice (Kasai et al. 2002) that described effects in the nose in both sexes at a chloroform concentration of 12 ml/m^3^. These differences can be explained by the higher susceptibility of BDF1 mice.

In a 13-week study, female and male F344 rats were exposed to chloroform concentrations of 0, 2, 10, 30, 90 or 300 ml/m^3^ for 6 hours a day, on 7 or 5 days a week (Table 10, Greim 2000; Templin et al. 1996). A **NOAEC** of **10 ml/m^3^** was established for cell proliferation induced by chloroform in the **kidneys**. After continuous exposure to concentrations of 2 ml/m^3^ and above, slight damage to the Bowman’s glands, respiratory metaplasia (called olfactory metaplasia by the authors), mineralization of the basal lamina and generalized atrophy of the ethmoturbinates were observed in the nose. As a result, instead of a NOAEC, only a **LOAEC** of **2 ml/m^3^** can be derived for the effects of chloroform in the **nose** of **rats**.

##### 5.2.1.3 104-Week studies

In a study of carcinogenicity and chronic toxicity in the kidneys, groups of 50 male F344 rats were exposed by inhalation for 6 hours a day, on 5 days a week, for 104 weeks. Other groups were exposed to chloroform both by inhalation and via the drinking water. The estimated uptake of chloroform by inhalation and via the drinking water is shown in Table 4. The body weights were reduced in all exposed animals in comparison with the values determined in the respective control groups. Drinking water consumption was markedly reduced in all animals given chloroform with the drinking water. Mortality did not differ between the groups. Several of the incidences for pre-neoplastic and non-neoplastic changes (no other details) are shown in Table 4.

**Tab.4:** Histopathological findings in the kidneys of male F344 rats after 2-year exposure to chloroform (Nagano et al. 2006)

	Concentration in the drinking water [mg/l]							
	0				1000			
Inhaled concentration [ml/m^3^]	0	25	50	100	0	25	50	100
estimated uptake of chloroform [mg/kg body weight and day]	0	20	39	78	45	73	93	135
number of animals	50	50	50	50	49	50	50	50
**kidneys**								
atypical tubule hyperplasia	1	0	0	0	2	4	7^c)^	15^a)^,^b)^,^c)^
cytoplasmic basophilia	0	3	7^a)^	8^a)^	9^a)^	26^a)^,^b)^,^c)^	35^a)^,^b)^,^c)^	36^a)^,^b)^,^c)^
dilated tubular lumens	0	3	11^a)^	27^a)^	28^a)^	46^a)^,^b)^,^c)^	48^a)^,^b)^,^c)^	49^a)^,^b)^,^c)^
nuclear enlargement in the proximal tubules	0	0	6^a)^	33^a)^	0	34^a)^,^b)^,^c)^	47^a)^,^b)^,^c)^	50^a)^,^b)^,^c)^
CPN severity +	7	21^a)^	21^a)^	30^a)^	21^a)^	2^a)^,^b)^,^c)^	13^a)^,^b)^,^c)^	17^a)^,^b)^,^c)^
CPN severity 2+	16	15	16	10	11	1	2	1
CPN severity 3+	26	5	3	2	2	0	0	1

CPN: chronic progressive nephropathy

a), b), c) statistically significant at p ≤ 0.05 according to the chi-squared test

a) in comparison with the values of the untreated control group

b) in comparison with the values of the inhalation control group

c) in comparison with the values of the groups exposed only via inhalation

In addition, a high incidence of urinary glucose (> 80%) was found in the animals of the groups with combined exposure, while a lower incidence was determined in the animals that were exposed only by inhalation or only by oral administration (< 15%) (Nagano et al. 2006).

In an inhalation study, chloroform concentrations of 0, 5, 30 or 90 ml/m^3^ were given to groups of 50 male and 50 female BDF1 mice and concentrations of 0, 10, 30 or 90 ml/m^3^ to groups of 50 male and 50 female F344 rats for 6 hours a day, on 5 days a week, for 104 weeks (Yamamoto et al. 2002). In response to the acute toxicity observed in the preliminary studies, the concentrations were successively adjusted in the mice of the 30 and the 90 ml/m^3^ groups (see Table 5).

**Tab.5:** Exposure scheme for the mice of the 104-week inhalation study (Yamamoto et al. 2002)

	Concentration	
	30 ml/m^3^	90 ml/m^3^
weeks 1 and 2	5 ml chloroform/m^3^	5 ml chloroform/m^3^
weeks 3 and 4	10 ml chloroform/m^3^	10 ml chloroform/m^3^
weeks 5 and 6	30 ml chloroform/m^3^	30 ml chloroform/m^3^
weeks 7 to 104	30 ml chloroform/m^3^	90 ml chloroform/m^3^

The data for this carcinogenicity study were already published by Matsushima (1994) and discussed in detail in Greim (2000). Additional data for non-neoplastic changes in the liver and kidneys and for clinical chemistry and urinalysis were published subsequently by Yamamoto et al. (2002) (see Table 6 and Table 7).

**Tab.6:** Non-neoplastic findings in the kidneys and liver of mice and rats after 104-week exposure to chloroform (Yamamoto et al. 2002)

**BDF1 mice**		**Concentration [ml/m^3^]**			
		**0**	**5**	**30**	**90**
number of animals	♂	50	50	50	48
	♀	50	49	50	48
**liver**					
central necrosis	♂	0	0	0	3
	♀	1	0	1	2
focal necrosis	♂	1	2	6	2
	♀	0	0	2	3
fatty changes	♂	4	2	6	24**
	♀	0	0	0	6*
altered cell foci	♂	10	1**	1**	5
	♀	0	1	2	6*
**kidneys**					
nuclear enlargement: proximal tubules	♂	0	3	43**	42**
	♀	0	0	0	4
cytoplasmic basophilia					
+	♂	33	40	8**	9**
	♀	0	4	3	5*
2+	♂	7	1	36	34
	♀	0	0	0	2
3+	♂	0	0	2	0
	♀	0	0	0	0
atypical tubule hyperplasia	♂	0	0	11**	14**
	♀	0	0	0	0
necrosis of the proximal tubules	♂	0	0	1	2
	♀	1	0	0	0
**F344 rats**		**Concentration [ml/m^3^]**			
		**0**	**10**	**30**	**90**
number of animals	♂	50	50	50	50
	♀	50	49	50	49
**liver**					
altered cell foci	♂	11	16	16	18
	♀	15	9	20	26
**kidneys**					
nuclear enlargement: proximal tubules	♂	0	0	5*	32**
	♀	0	0	6*	34**
dilated tubular lumen	♂	0	0	9*	27**
	♀	0	0	5*	38**
chronic progressive nephropathy					
+	♂	3	11*	10**	17**
	♀	8	19**	27**	15**
2+	♂	6	10	24	14
	♀	15	7	5	3
3+	♂	19	15	8	2
	♀	14	3	3	1
4+	♂	19	8	2	1
	♀	4	2	0	2

*p ≤ 0.05;

**≤ 0.01 according to the chi-squared test

Severity of cytoplasmic basophilia: +: few lesions in individual tubules, 2+: more than four lesions in two or more tubules, 3+: multiple lesions within the entire section

Severity of nephropathy: +: mild, 2+: moderate, 3+: marked, 4+: severe

The Commission received a personal communication from the authors (Nagano and Matsumoto 2019) relating to the histopathological findings in the nasal cavity. The authors did not calculate the statistical significance of the effects in the nose (see Table 8).

In **mice**, alkaline phosphatase (ALP) in the serum was increased with statistical significance after exposure to chloroform concentrations of 5 ml/m^3^ and above for 104 weeks, which may be an early sign of liver damage. At a chloroform concentration of 90 ml/m^3^, the incidences of fatty changes in the livers of male and female mice and of altered cell foci in female mice were increased with statistical significance. In male mice, nuclear enlargement in the proximal tubules was observed in the kidneys; the first signs of this effect became evident at concentrations of 5 ml/m^3^ and above and the incidences were increased with statistical significance at 30 ml/m^3^ and above. In addition, atypical tubule hyperplasia was observed in the male animals at concentrations of 30 ml/m^3^ and above. Blood urea nitrogen was decreased in the male mice at chloroform concentrations of 30 ml/m^3^ and above, suggesting impairment of the kidneys.

In male and female mice, high incidences of slight respiratory metaplasia in the olfactory epithelium (43% of the exam­ined animals) and slight bone thickening (75% of the examined animals) developed at chloroform concentrations of 5 ml/m^3^ and above. Comparison with the effects found in the next-higher concentration group (30 ml chloroform/m^3^) revealed a dose-response relationship for the effects in the olfactory epithelium of male mice. Compared with the values determined in the concentration group exposed to 5 ml/m^3^, lower incidences of bone thickening in male mice and of all effects in the nose of female mice were observed at a chloroform concentration of 30 ml/m^3^.

**Tab.7:** Findings of the haematological examination and urinalysis in mice and rats after 104-week exposure to chloroform (Yamamoto et al. 2002)

BDF1 mice		Concentration [ml/m^3^]			
		0	5	30	90
number of animals	♂	33	38	36	35
	♀	28	34	24	24
**clinical chemistry**					
total protein [g/dl]	♂	5.6	5.8	5.9	6.1**
	♀	5.5	5.5	5.6	6.0
triglycerides [mg/dl]	♂	83	82	101	80
	♀	82	72	81	58**
BUN [mg/dl]	♂	25.8	22.5	26.3*	30.8**
	♀	17.2	21.4	19.3	21.4**
GOT [IU/l]	♂	85	58	115	111*
	♀	144	148	125	175*
GPT [IU/l]	♂	29	16	40	44**
	♀	28	47	39	50**
ALP [IU/l]	♂	171	184*	219**	205**
	♀	264	322	235	303
**F344 rats**		**Concentration [ml/m^3^]**			
		**0**	**10**	**30**	**90**
number of animals	♂	27	39	36	38
	♀	37	35	40	34
**clinical chemistry**					
total protein [g/dl]	♂	6.7	7.1	7.0	6.9
	♀	7.0	7.4*	7.3	7.2
total cholesterol [mg/dl]	♂	173	164	153	125**
	♀	142	131	135	149
triglycerides [mg/dl]	♂	222	167	146*	87**
	♀	191	126	109	94**
phospholipids [mg/dl]	♂	289	268	241*	196**
	♀	280	252	255	271
creatinine [mg/dl]	♂	0.9	0.6	0.6**	0.7**
	♀	0.5	0.5	0.5	0.5
BUN [mg/dl]	♂	28.6	20.6**	18.3**	23.2**
	♀	17.9	18.5	18.5	18.6
GOT [IU/l]	♂	67	79	81	98*
	♀	128	113	124	144
GPT [IU/l]	♂	21	25*	24	26*
	♀	37	40	39	48
gamma-GT [IU/l]	♂	4	8*	10**	7*
	♀	4	4	5	6**
LDH [IU/l]	♂	164	239	179	311
	♀	297	245	239*	344
**urinalysis**					
glucose^a)^	♂	0/27	0/39	2/37	29/39**
	♀	2/41	8/37*	29/41**	24/34**

* p ≤ 0.05

** p ≤ 0.01 in Dunnett’s test for clinical chemistry and the chi-squared test for urinalysis

a) number of animals with positive results in the glucose test/total number of tested animals

ALP: alkaline phosphatase; BUN: blood urea nitrogen; gamma-GT: gamma-glutamyltransferase; GOT: glutamate oxaloacetate transaminase; GPT: glutamate pyruvate transaminase; LDH: lactate dehydrogenase

**Tab.8:** Non-neoplastic changes in the nasal cavity of mice and rats after 104-week exposure to chloroform (Nagano and Matsumoto 2019)

**BDF1 mice**		**Concentration [ml/m^3^]**			
		**0**	**5**	**30**	**90**
number of animals	♂	50	50	50	48
	♀	50	49	50	48
**nasal cavity**					
bone thickening					
slight	♂	0	49	37	41
	♀	0	37	32	33
moderate	♂	0	0	0	1
	♀	0	0	0	1
marked	♂	0	0	0	0
	♀	0	0	0	0
severe	♂	0	0	0	0
	♀	0	0	0	0
**respiratory metaplasia in the olfactory epithelium**					
slight	♂	12	15	20	21
	♀	4	21	14	25
moderate	♂	0	0	0	0
	♀	0	0	0	0
marked	♂	0	0	0	0
	♀	0	0	0	0
severe	♂	0	0	0	0
	♀	0	0	0	0
**F344 rats**		**Concentration [ml/m^3^]**			
		**0**	**10**	**30**	**90**
number of animals	♂	50	50	50	50
	♀	50	49	50	49
**nasal cavity**					
bone thickening					
slight	♂	0	35	44	33
	♀	0	39	40	25
moderate	♂	0	0	0	5
	♀	0	0	3	3
marked	♂	0	0	0	0
	♀	0	0	0	0
severe	♂	0	0	0	0
	♀	0	0	0	0
**respiratory metaplasia in the olfactory epithelium**					
slight	♂	0	29	19	8
	♀	0	29	27	15
moderate	♂	0	11	22	16
	♀	0	14	10	11
marked	♂	0	2	7	19
	♀	0	0	10	17
severe	♂	0	0	2	0
	♀	0	0	1	1

significance not calculated

No statistically significant changes were observed in the liver in female and male **rats**. Glutamate-pyruvate trans­aminase and gamma-glutamyltransferase levels were increased with statistical significance, but not with a clear concentration–effect relationship. As liver damage was found in many of the animals in the control group, the liver effects in the lower concentration range are difficult to evaluate and it is questionable that they were caused by the substance. In female rats, glucose levels in the urine were increased at chloroform concentrations of 10 ml/m^3^ and above. This suggests that less glucose was taken up via the renal tubules and that the kidneys were damaged. A typical finding in rats, chronic progressive nephropathy (CPN), was observed in both sexes at chloroform concentrations of 10 ml/m^3^ and above. This effect is not important for the evaluation because it is not considered relevant to humans (Hard et al. 2009). At chloroform concentrations of 30 ml/m^3^ and above, a statistically significant increase in nuclear enlargement in the proximal tubules and dilation of the tubular lumen were observed in female and male rats. These effects were not found in the control animals.

No findings in the nose were reported for the rats of the control groups. In contrast, high incidences of slight bone thickening and respiratory metaplasia in the olfactory epithelium were detected in both sexes at chloroform concentrations of 10 ml/m^3^ and above (Nagano and Matsumoto 2019; Yamamoto et al. 2002).

In this **104-week study**, effects on the **nose** were the most sensitive end point in rats and mice because of the high incidence of slight respiratory metaplasia in the olfactory epithelium and slight bone thickening. As these effects already occurred at the lowest concentration tested, it is not possible to derive a NOAEC, but only a LOAEC, for both species. Therefore, the **LOAEC** for **rats** was determined to be **10 ml chloroform/m^3^** and the** LOAEC** for **mice 5 ml chloroform/m^3^**.

It is noteworthy that bone thickening in the nose of rats and mice was already present in a high incidence at the lowest concentration tested; however, this effect did not increase in severity with the concentration. The pathomechanism of bone thickening is explained by the degeneration of the Bowman’s glands, leading to irritation of the periosteum and subsequent proliferation of the bone cells. Rats are described as particularly susceptible to damage to the Bowman’s glands (see Section 3.2). On the basis of these findings, rats are more susceptible to the induction of bone thickening than humans.

In mice, a concentration–effect relationship was not established for the respiratory metaplasia, which is caused by the degeneration of the Bowman’s glands, but also by irritation alone. In rats, the severity increased with the concentration.

##### 5.2.1.4 Overall summary

The relevant results of the histopathological examination of the liver, kidneys and nose of mice and rats after inhalation exposure to chloroform for 13 and 104 weeks are compared in Tables 9 and 10. These studies identified the nose as the most sensitive organ in both species. In **rats**, a LOAEC of 2 ml/m^3^ was established for chloroform after exposure for 13 weeks (Templin et al. 1996). The observed effects were slight damage to the Bowman’s glands, respiratory metaplasia in the olfactory epithelium, mineralization of the basal lamina and generalized atrophy of the ethmoturbinates. When evaluating these findings, it must be taken into account that the animals were exposed continuously without a recovery period. A comparison of the findings of studies with exposure intervals of 5 days a week and those with exposure for 7 days a week showed that the effects were always more severe after continuous exposure. Therefore, studies using the exposure interval of 5 days a week that is more relevant to the workplace are better suited for the derivation of an occupational exposure limit value. The 2-year study that determined a **LOAEC** for chloroform of **10 ml/m^3^** for **rats** and of **5 ml/m^3^** for **mice** is likewise relevant (Yamamoto et al. 2002). Respiratory metaplasia and bone thickening in the nose of rats and mice were observed in this study. Respiratory metaplasia is more relevant to the evaluation as humans seem to be less susceptible to bone thickening (Section 3.2). Metaplasia is more plausible for rats than for mice because there is a recognizable concentration–effect relationship.

**Tab.9:** Findings of the histopathological examination of the liver, kidneys and nose of mice after inhalation exposure to chloroform for 13 weeks and 104 weeks

Concentration	Duration Animal strain References			
	13 weeks, 5 days/week B6C3F1 mice Larson et al. 1996	13 weeks, 7 days/week B6C3F1 mice Larson et al. 1996	13 weeks, 5 days/week BDF1 mice Kasai et al. 2002	104 weeks, 5 days/weekBDF1 miceNagano and Matsumoto 2019^a)^, Yamamoto et al. 2002
0 ml/m^3^	**liver**: swelling and vacuolization of the centrilobular hepatocytes (♀ 1/15, S: 1.0; ♂ 5/15, S: 1.0), **kidneys**: no findings, **nose**: no findings	**liver**: swelling and vacuolization of the centrilobular hepatocytes (♀ 1/15, S: 1.0; ♂ 5/15, S: 1.08), **kidneys**: no findings, **nose**: no findings	**liver, kidneys, nose**: no findings	**liver**: ♀ central necrosis (1/50), ♂ fatty changes (4/50), altered cell foci (10/50), focal necrosis (1/50), **kidneys**: ♀ tubular necrosis (1/50), ♂ cytoplasmic basophilia (40/50), **nose**: slight respiratory metaplasia in the olfactory epithelium (♀: 4/50, ♂: 12/50)
0.3 ml/m^3^	not examined	**liver**: swelling and vacuolization of the centrilobular hepatocytes (♀ 1/15, S: 1.0; ♂ 4/15, S: 1.5)	not examined	not examined
2 ml/m^3^	not examined	**liver**: **NOAEC**, swelling and vacuolization of the centrilobular hepatocytes (♀ 5/14, S: 1.4; ♂ 5/14, S: 1.0), **kidneys**: no findings, **nose**: no findings	not examined	not examined
5 ml/m^3^	not examined	not examined	not examined	**liver**: **NOAEC**, ♀ altered cell foci (1/49), ♂ fatty changes (2/50), altered cell foci (1/50), focal necrosis (2/50), **kidneys: NOAEC**, cytoplasmic basophilia (♀ 4/49; ♂ 41/50), ♂ cell growth in the proximal tubules (3/50), **nose**: **LOAEC**, slight respiratory metaplasia in the olfactory epithelium (♀ 21/49; ♂ 15/50), slight bone thickening (♀ 37/49; ♂ 49/50)
10 ml/m^3^ and 12 ml/m^3^ (Kasai et al. 2002)	**liver**: **LOAEC**, swelling and vacuolization of the centrilobular hepatocytes (♀ 2/13, S: 1.0; ♂ 4/13, S: 1.5), **kidneys**: **LOAEC**, ♂ nephropathy (1/13, S: 1.0), enlarged nuclei in the proximal tubule cells in the renal cortex, significant increase in cell proliferation in the cortex (labelling index: 7.6 ± 3.0; controls: 1.6 ± 0.6) and medulla (labelling index: 1.6 ± 0.8; controls: 0.8 ± 0.3), **nose**: no findings	**liver**: swelling and vacuolization of the centrilobular hepatocytes (♀ 4/14, S: 2.0; ♂ 5/15, S: 1.0), **kidneys**: ♂ enlarged nuclei in the proximal tubule cells in the renal cortex, **nose**: no findings	**liver**: no findings, **kidneys**: **LOAEC**, statistically significant tubular necrosis (♂ 7/10), degeneration (♂ 2/10), significant cytoplasmic basophilia (♂ 8/10), **nose**: **LOAEC**, degeneration of the olfactory epithelium (♂ 2/10), statistically significant bone thickening (♂ 7/10; ♀ 7/10), statistically significant eosinophilic changes in the olfactory epithelium (♀ 7/10) and in the respiratory epithelium (♀ 8/10)	not examined
25 ml/m^3^ (Kasai et al. 2002) and 30 ml/m^3^	not examined	**liver**: ♀ swelling and vacuolization of the centrilobular hepatocytes (10/15, S: 1.2), ♂ like ♀ (12/12, S: 1.2), **kidneys**: ♂ nephropathy (11/12, S: 1.0), enlarged nuclei in the proximal tubule cells in the renal cortex, statistically significant increase in cell proliferation in the cortex (labelling index: 2.4 ± 1.2; controls: 0.8 ± 0.3), **nose**: no findings	**liver**: no findings, **kidneys**: significant tubular necrosis (♂ 10/10), significant degeneration (♂ 9/10), cytoplasmic basophilia (♂ 1/10), **nose**: statistically significant degeneration of the olfactory epithelium (♂ 5/10), bone thickening (♂ 1/10; statistically significant ♀ 10/10), statistically significant eosinophilic changes in the olfactory epithelium (♀ 9/10) and in the respiratory epithelium (♀ 8/10)	**liver**: ♀ central and focal necrosis (1/50 and 2/50, respectively), altered cell foci (2/50), ♂ fatty changes (6/50), altered cell foci (1/50), focal necrosis (6/50), **kidneys**: cytoplasmic basophilia (♀ 3/50; ♂ 46/50), ♂ statistically significant increase in nuclear enlargement in the proximal tubules (43/50), statistically significant increase in atypical tubule hyperplasia (11/50), tubular necrosis (1/50), **nose**: slight respiratory metaplasia in the olfactory epithelium (♀ 14/50; ♂ 20/50), slight bone thickening (♀ 32/50; ♂ 37/50)

a) statistical significance not calculated

S: Severity on a scale of 1: minimal, 2: slight, 3: moderate, 4: severe

**Tab.10:** Findings of the histopathological examination of the liver, kidneys and nose of rats after exposure to chloroform for 13 weeks and 104 weeks

Concentration	Duration Animal strain References			
	13 weeks, 5 days/week F344 rats Templin et al. 1996	13 weeks, 7 days/week F344 rats Templin et al. 1996	13 weeks, 5 days/week F344 rats Kasai et al. 2002	104 weeks, 5 days/week F344 rats Nagano and Matsumoto 2019a); Yamamoto et al. 2002
0 ml/m^3^	**liver**: damage (♀ Sb): 0.1; 1/15; ♂ Sb): 0.1; 1/15), **kidneys**: damage (♀ Sb): 0.4; 6/14; ♂ Sb): 0.6; 8/14), **nose**: no findings	**liver**: damage (♀ Sb): 0.1; 1/15; ♂ Sb) 0.1; 1/15), **kidneys**: damage (♀ Sb): 0.4; 6/14; ♂ Sb): 0.6; 8/14), **nose**: no findings	**liver, kidneys, nose**: no findings	**liver**: altered cell foci (♀ 15/50; ♂ 11/50), **kidneys**: no findingsc), **nose**: no findings
2 ml/m^3^	not examined	**liver**: damage (♀ Sb): 0.1; 1/15; ♂ Sb): 0.2; 3/15), **kidneys**: damage (♀ Sb): 0.7; 10/15; ♂ Sb): 0.8; 10/15), **nose**:** LOAEC**, damage (♂ Sb): 1.1; 10/10), ♀ like ♂ (no other data)	not examined	not examined
10 ml/m^3^	not examined	**liver**: no findings, **kidneys**: **NOAEC**, damage (♀ Sb): 0.7; 10/15; ♂ Sb): 0.5; 7/15), **nose**: damage (♂ Sb): 2.0; 10/10), increased cell proliferation* (ULLI: 4 times as high as the control value), ♀ like ♂ (no other data)	not examined	**liver**: altered cell foci (♀ 9/49; ♂ 16/50), **kidneys**: **NOAEC**, **nose**:** LOAEC**, slight bone thickening (♀ 39/49, ♂ 35/50), respiratory metaplasia in the olfactory epithelium (♀ slight 29/49, moderate 14/49; ♂ slight 29/50, moderate 11/50, marked 2/50)
25 ml/m^3^ (Kasai et al. 2002) and 30 ml/m^3^	**liver**: no findings, **kidneys**: **LOAEC**, **♀ **damage (Sb): 1.8; 13/13), no statistically significant increase in cell proliferation in cortex (labelling index: 2 times as high as the control value), ♂ damage (Sb): 0.1; 2/15), **nose**: **LOAEC**, ♂ damage (Sb): 1.8; 8/8), increased cell proliferation* (ULLI: 2 times as high as the control value), ♀ like ♂ (no other data)	**liver**: damage (♂ Sb): 0.1; 2/15), **kidneys**: damage (♀ Sb): 0.8; 12/15, ♂ Sb): 0.6; 9/14), increased cell proliferation* in the cortex (♀ labelling index: 3 times as high as the control value; ♂ labelling index: 2 times as high as the control value), **nose:** damage (♂ Sb): 2.0; 10/10), increased cell proliferation* (ULLI: 4 times as high as the control value), ♀ like ♂ (no other data)	**liver**: no findings, **kidneys**: no findings, **nose**:** LOAEC**, statistically significant mineralization (♀ 10/10**; ♂ 10/10**), statistically significant atrophy of the olfactory epithelium (♀ 10/10**; ♂ 7/10**)	**liver**: altered cell foci (♀ 20/50; ♂ 16/50), **kidneys**: ♀ nuclear enlargement in the proximal tubules (♀ 6/50*; ♂ 5/50*), dilation of the tubular lumen (♀ 5/50*; ♂ 9/50*), **nose**: bone thickening (♀ slight 40/50, moderate 3/50; ♂ slight 44/50), respiratory metaplasia in the olfactory epithelium (♀ slight 27/50, moderate 10/50, marked 10/50, severe 1/50; ♂ slight 19/50, moderate 22/50, marked 7/50, severe 2/50)

* p ≤ 0.05

** p ≤ 0.01

a) statistical significance not calculated

b) Mean of the entire group of affected animals. Kidneys: vacuolization of the proximal tubular epithelial cells, enlarged proximal tubular cell nuclei, pyknotic (concentrated) proximal tubular cell nuclei as well as isolated tubular cell necrosis; liver: vacuolization of the hepatocytes, degenerative changes in the hepatocytes and necrosis of the hepatocytes; nose: damage to the Bowman’s glands ranging from oedema to loss of the glands, olfactory metaplasia, mineralization of the basal lamina, generalized atrophy of the ethmoturbinates

c) no findings except for chronic progressive nephropathy, which is not relevant to humans

S: Severity of damage on a scale of 0: within the normal limits, 1: minimal, 2: slight, 3: moderate, 4: severe; ULLI: unit length labelling index

#### 5.2.2 Oral administration

The effects induced by chloroform after oral administration are described in detail in Greim (2000). In rats, a NOAEL (no observed adverse effect level) of 3.5 mg/kg body weight and day was determined after exposure to chloroform in the drinking water for 3 weeks; in mice, the NOAEL was 10 mg/kg body weight and day after exposure by gavage. When evaluating the findings of inhalation studies, it is important to consider that lesions in the nose were observed in rats given gavage doses of chloroform of 34 mg/kg body weight and day (Greim 2000; Larson et al. 1995). There are no relevant new data available.

### 5.3 Local effects on skin and mucous membranes

#### 5.3.1 Skin

Chloroform causes irritation of the skin of rabbits (Greim 2000). There are no relevant new data available.

#### 5.3.2 Eyes

Chloroform causes irritation of the eyes of rabbits (Greim 2000). There are no relevant new data available.

#### 5.3.3 Allergenic effects

The ECHA database includes a local lymph node assay (LLNA) in female CBA/J mice that obtained negative results with a 10% preparation in acetone/olive oil (4:1). A stimulation index of 2.48 was determined for this concentration (ECHA 2020).

A maximization test that can likewise be found in the ECHA database cannot be included in the evaluation because similar responses were produced by the 5 animals given undiluted chloroform for intradermal and topical induction and by the 3 control animals (ECHA 2020).

An earlier study reported positive results for an LLNA carried out with a chloroform/methanol mixture (2:1). After the undiluted mixture was applied 3 times, stimulation indices of 8.5 and 10.2, respectively, were determined in 2 experiments. However, this study likewise obtained positive results with several other irritant substances (e. g. oxalic acid and sodium lauryl sulfate) (Montelius et al. 1994).

### 5.4 Reproductive and developmental toxicity

#### 5.4.1 Fertility

A multigeneration study with ICR mice given oral doses of chloroform is described in detail in the addendum from 1999 (Greim 2000). In the high dose group that was given a chloroform concentration of 5 mg/ml with the drinking water (equivalent to a chloroform dose of 1000 mg/kg body weight and day assuming the consumption of 6 ml of drinking water a day and a body weight of 30 g), statistically significant effects on reproduction such as reduced fertility, litter size, gestation index and viability index were observed in all F1 and F2 animals. At the same time, there was an increase in mortality and a delay in body weight gains (WHO 1994).

Three other studies that investigated fertility are available that were not yet included in the addenda and are therefore presented below.

In a continuous mating study, male and female CD-1 mice (20 pairs per dose group, 40 pairs in the control group) were given chloroform in gavage doses of 6.6, 16 or 41 mg/kg body weight and day. Corn oil was used as the solvent and the control substance. The F0 animals were exposed daily for a total of 18 weeks (1 week before mating, 14 weeks during mating and 3 weeks after mating). The F0 animals were examined for clinical signs, and the body weights, drinking water consumption, fertility (number of pairs producing a litter/number of breeding pairs), litter size, number of live pups per litter and distribution of the surviving offspring were determined. The sex and weight of the offspring were determined directly after birth. In the F1 generation, only the animals of the control group and the high dose group continued to be treated like their parents. The first dose was administered to the F1 animals on postnatal day 22 after the last litter was born and the animals had been weaned. Male and female F1 animals from different litters of the same treatment group were mated for 7 days. The animals of the F1 generation were examined for the same fertility end points as the F0 animals. After birth, the animals of the F1 generation were sacrificed and the organ weights, body weights, sperm motility in the epididymis, sperm morphology, sperm count and oestrus cycle were examined. In addition, the testes, epididymis, ovaries and the liver, lungs, kidneys and the thyroid gland were examined by pathological examination. No substance-induced effects were observed in the F0 generation. The following effects were found in the F1 animals that were exposed to chloroform: a statistically significant increase in body weights was observed in the female animals from week 31 onwards and the fertility index, number of female offspring per litter and the overall litter size were increased with statistical significance. In the females, the absolute and relative liver weights were increased with statistical significance and accentuated lobular patterns were detected in the livers of 13 animals (1 control animal). Centrilobular degeneration in the hepatocytes was observed in all female animals. In the male animals, a statistically significant increase in the weights of the right epididymis (absolute and relative) was determined and, in 8 of 20 animals (3 of 20 animals of the control group), minimal to slight vacuolar degeneration in the epithelium of the epididymal duct. The NOAEL for fertility was 41 mg/kg body weight and day, the highest dose tested (NTP 1988).

In a modified multigeneration study, chloroform was given to 10 male and 30 female ICR Swiss mice with the drinking water in concentrations of 0.1, 1 or 5 mg/ml. After 35 days, the mice were mated for 7 days, with 1 of the male F0 mice paired with 3 of the female F0 mice. Two weeks after weaning of the F1a offspring, the F0 mice were mated to produce the F1b generation. The F1c generation was produced according to the same scheme. Female mice of the F1b and F0 generations were likewise mated with untreated male mice to produce a F2 generation for an integrated teratogenicity study. With the exception of the F1b generation, necropsy was performed on all offspring and on all adult animals after weaning. Significant maternal toxicity was found in the high dose groups of the F0 and F1b generations. Effects on fertility, litter size, the gestation index and postnatal survival were observed only in the high dose group and concurrently with maternal toxicity. No statistically significant effects were observed in the teratogenicity study (Borzelleca and Carchman 1982). The study was not carried out according to the standards valid today and the description of methods and results has considerable shortcomings.

Groups of 5 (C57Bl/C3H)F1 mice were exposed by inhalation to chloroform concentrations of 0, 400 or 800 ml/m^3^ for 4 hours a day, on 5 days. The morphology of the epididymal sperm was examined 28 days after exposure. After inhalation of chloroform, the number of abnormal sperm was increased with statistical significance (3.48 ± 0.66 and 2.76 ± 0.31, respectively) (Land et al. 1981). The number of sperm and the fraction of mobile sperm were not investigated. As a result, the study has not been included in the evaluation.

**Summary: **Effects on fertility occurred only at high chloroform doses of about 1000 mg/kg body weight and day and concurrently with maternal toxicity.

#### 5.4.2 Developmental toxicity

Studies investigating developmental toxicity are described in detail in the chapter “MAK values and pregnancy” published in 1989 and in the addendum from 1999 (Greim 2000). No new information has become available.

In two prenatal developmental toxicity studies (one carried out according to OECD Test Guideline 414) with whole-body exposure of Wistar rats to chloroform, developmental delays in the form of reduced body weights and reduced crown–rump lengths were found at concentrations of 30 ml/m^3^ and above. At the same time, maternal toxicity including reduced body weight gains and reduced feed consumption was already evident. The NOAEC for developmental toxicity was 10 ml/m^3^ (Hoechst AG 1988, 1990 a, b). The NOAEC for developmental toxicity of 10 ml/m^3^ was confirmed by assessing the original studies.

In a valid study with whole-body exposure of a different rat strain (Sprague Dawley), external malformations including shortened or missing tails, imperforate anuses and skeletal malformations (missing ribs) were observed in 3 litters at a concentration of 100 ml/m^3^. The NOAEC for these effects was 30 ml/m^3^ (Schwetz et al. 1974). This study is regarded as valid. In the foetuses, reduced body weights per litter and a slightly shortened crown–rump length were determined at 30 ml/m^3^; however, no concentration dependency was found for these effects. The litters at this concentration were the largest in size; this may explain the slightly shorter crown–rump lengths. Malformations were observed in 3 litters at 100 ml/m^3^; these are regarded as evidence of teratogenicity.

### 5.5 Genotoxicity

The data for genotoxicity are described in detail in the addenda from 1999 and 2003 (Greim 2000; Hartwig 2010). The present addendum briefly reviews the data available to date and evaluates new studies. Studies published prior to 2003 that were not included in the earlier addenda are discussed in detail only if their findings contradict the previous evaluation results.

#### 5.5.1 In vitro

In a rec-assay with Bacillus subtilis that was already included in Greim (2000), negative results were obtained with chloroform in the presence of a metabolic activation system (Kada 1981). Another assay that accounted for the volatility of the test substance in its study design yielded positive results (Matsui et al. 1989). Important information was not reported, such as the chloroform concentration at which the damage became evident or data for the purity of the test substance. As a result, the validity of this study is greatly limited.

A test for the induction of an SOS response using the Salmonella typhimurium strain TA1536/pSK1002 (umu test) was carried out after incubation with chloroform for 2, 4, 6 and 20 hours both in the presence and absence of a metabolic activation system. A chloroform concentration of 1000 mg/l yielded positive findings after 6 hours without the addition of S9 mix as well as after 20 hours with the addition of metabolic activation. There are no data for purity and for the solvent used (Ono et al. 1991). Positive results were obtained with chloroform only at very high concentrations. However, based on the examined growth curves, it is assumed that the concentration that triggered the SOS response also induced strong cytotoxic effects.

To date, chloroform has not been found to be mutagenic in bacteria (Greim 2000). This is confirmed by negative results in the Salmonella typhimurium strains TA98, TA100, TA1535, TA1537 and in the Escherichia coli strain WP2uvrA/pKM101. However, after the addition of a metabolic activation system and GSH, a slight, yet statistically significant increase in the number of revertant colonies was found in the Escherichia coli strain WP2/pKM101 at chloro­form concentrations of 0.05% to 2%. The number of colonies was up to 2.1 times as high as the control number. This increase was not observed without the addition of GSH. The highest concentration of 5% was cytotoxic. The study used gas-phase exposure with a gas sampling bag to prevent the dissipation of chloroform (Araki et al. 2004). The Escherichia coli strain WP2/pKM101 is used to detect mutations at an A:T base pair and, unlike WP2uvrA/pKM101, is competent for excision repair. The use of this strain and the Salmonella typhimurium strain TA102 is recommended by OECD Test Guideline 471 for the detection of DNA cross-linking or oxidizing substances. In this study, ethanol was used in concentrations of 0.3% to 1% to stabilize chloroform. Adding ethanol to chloroform may lead to the formation of the potent alkylating agents ethyl or diethyl carbonate and thus have an effect on the results (US EPA 2001). A different study that tested chloroform concentrations of 0.1 to 1000 µg/plate with the Escherichia coli strain WP2/pKM101 yielded negative results in the presence and in the absence of a metabolic activation system (Kirkland et al. 1981).

Chloroform was strongly mutagenic in the Salmonella typhimurium strains TA98, TA100, TA1535 and TA1537 at concentrations of 0.2 µmol/plate without the addition of metabolic activation. In the presence of a metabolic activation system, the number of revertant colonies and thus mutagenicity were much lower (Varma et al. 1988). This study likewise used ethanol, which may have influenced the results (see above). Mutagenicity was higher without metabolic activation than with metabolic activation. This does not seem consistent with the mechanism of action, as toxicity ­occurs after metabolic activation. However, substances may bind to the metabolic activation system, thereby interfering with the uptake in the cell. Furthermore, positive findings were observed only at the lowest concentration both with and without the addition of metabolic activation. Therefore, no concentration–effect relationship was evident. Overall, the results are not plausible.

Negative results were obtained in yeast-based genotoxicity tests (Kassinova et al. 1981; Mehta and von Borstel 1982; Scheel and Zimmermann 1981). In a test for the induction of deletions (DEL) via intrachromosomal recombination in Saccharomyces cerevisiae, chloroform yielded positive results at concentrations of 0.75 to 5.6 mg/ml. The DEL recombination frequency was increased 5.6-fold. The addition of *N*-acetylcysteine (NAC) as a free radical scavenger reduced the induction of DEL recombination (except at the high concentration) and resulted in a lower level of cyto­toxicity. In addition, chloroform oxidized dichlorofluorescein diacetate, a marker for free radicals, suggesting that chloroform is able to generate free radicals within the cell (Brennan and Schiestl 1998). Cell death was determined even at the lowest concentration tested; with the addition of NAC a maximum of 64% of the cells survived, without the addition of NAC 52% survived. Therefore, all results were obtained under cytotoxic conditions. A metabolic activation system was not used.

Chloroform was found to have mutagenic and clastogenic potential in eukaryotic cells only at cytotoxic concentrations (Greim 2000; Hartwig 2010). A recent study investigated whether chloroform leads to the induction of secondary genotoxicity in the hepatocytes of female Wistar rats by means of oxidative stress. Cytotoxicity was determined after 2 hours based on the release of lactate dehydrogenase and became evident only at 20 mM chloroform and above. In addition, a significant increase in cytotoxicity was determined by MTT (3-[4,5-dimethylthiazol-2-yl]-2,5-diphenyltetrazolium bromide) test at 8 mM and above (activity reduced by 12%). The GSH level was reduced with statistical significance only at cytotoxic concentrations of 20 mM. After incubation with chloroform for 2 hours, a statistically significant increase in DNA single strand breaks was determined by comet assay only at concentrations of 8 mM and above. 8-Oxodeoxyguanosine levels were analysed after incubation for 2 hours with chloroform concentrations of 4, 8 or 20 mM and were not increased with statistical significance. Lipid peroxidation was determined by means of malondialdehyde and 4-hydroxynonenal. At concentrations of 4 mM and above and an incubation period of 2 hours, a statistically significant increase in lipid peroxidation products and a concentration-dependent, statistically significant increase in malondialdehyde deoxyguanosine (M1dG) adducts were observed (Beddowes et al. 2003). The data given by the authors for the onset of cytotoxicity are contradictory. An MTT assay is described, but not included as a figure, that yielded findings of significant cytotoxicity at concentrations of 8 mM and above. According to the description of the assay, single strand breaks were observed with the onset of cytotoxicity. The GSH levels were not reduced and the 8-oxo-deoxyguanosine levels were not increased; this is regarded as evidence of genotoxicity that is not mediated by ROS. However, the assessment of the GSH status did not include a determination of GSSG, which would have shown the induction of GSH formation by GSSG. As a result, a possible decrease in GSH may have been masked.

V79 cells were incubated with chloroform concentrations of 6, 10 or 12 mM and then examined for aneuploidy. Chloroform led to a disturbance in the spindle apparatus, in some cases at cytotoxic concentrations (Önfelt 1987).

#### 5.5.2 In vivo

According to the previously available data, chloroform did not induce mutagenic effects in the somatic or germ cells of Drosophila melanogaster. Negative results were obtained with transgenic lacI mice for gene mutations induced by chloroform in the liver. The results obtained by in vivo genotoxicity tests with mammals suggest a clastogenic potential, but only at high, mostly toxic doses (Greim 2000; Hartwig 2010). A study has now investigated the clastogenic and aneugenic potential of chloroform. Another study investigated the DNA binding activity of chloroform. These two recent studies are discussed below.

In a micronucleus test in the erythrocytes of the bone marrow carried out according to OECD Test Guideline 474, groups of 6 male and 6 female Sprague Dawley rats were given gavage doses of chloroform of 120, 240 or 480 mg/kg body weight and day on 5 consecutive days. Corn oil was used as the solvent control, cyclophosphamide as the clastogenic positive control and carbendazim as the aneugenic positive control. All animals were sacrificed 24 hours after treatment with the last dose. In the high dose group given 480 mg/kg body weight and day, clinical signs were observed including impaired coordination of movements, decreased and increased breathing rates, bent posture, reduced body temperature, lethargy, nose rubbing, reduced activity, ptosis, piloerection and tremor. A dose-dependent reduction in body weight gains was found in the exposed animals in comparison with the findings in the solvent control group. The positive controls and the solvent controls yielded the expected results. The ratio of polychromatic and normochromatic erythrocytes, expressed as the percentage of polychromatic erythrocytes, decreased with the dose and was 38% (♂) and 27% (♀) in the 480 mg/kg group and 62% (♂) and 44% (♀) in the control group. This was regarded by the authors as a sign of toxic effects in the bone marrow and that the target organ was reached. When evaluated in conjunction with the clinical signs, these findings show that the doses tested were sufficiently high. Of 2000 counted polychromatic erythrocytes, the individual fraction with micronuclei was of the same order of magnitude as that of the solvent control. The mean number of polychromatic erythrocytes with micronuclei found in each dose group did not differ with statistical significance from the mean number determined in the control group. Overall, the study demonstrates that chloroform is neither clastogenic nor aneugenic in rats at up to 480 mg/kg body weight and day (Covance Laboratories Ltd 2009).

In a study investigating the covalent binding of chloroform to the DNA, RNA and proteins, radioactively-labelled [U-14C]-chloroform was given to 6 male Wistar rats and male BALB/c mice by intraperitoneal injection. The dose was 500 µCi/kg body weight. The animals were sacrificed after 22 hours; the liver, lungs, kidneys and stomach were removed and examined for bound activity. In addition, mouse liver DNA that was enzymatically digested to 5′-mono­nucleotides was examined by HPLC for bound activity. In another experiment, 6 rats and 18 mice were pre-treated intraperitoneally for enzyme induction with phenobarbital or beta-naphthoflavone 2 days before they were given chloroform by injection. In both species, binding to the RNA and to proteins was higher than binding to the DNA. According to the authors, this suggests that chloroform has weak DNA binding activity. With the exception of the stomach, higher DNA binding activity was observed in the organs of mice than in those of rats. An HPLC examination found that all of the covalently bound radioactivity was determined in a single peak and did not coincide with the peaks of the unmodified nucleotides. This was regarded as evidence that a DNA adduct was induced by chloroform, but without identifying a specific adduct. Pre-treatment with the CYP450 inducers phenobarbital or beta-naphtho­flavone did not change the binding activity of chloroform to the DNA in the liver of mice with statistical significance, but decreased this activity in the liver of rats (Colacci et al. 1991). The DNA adduct was not identified in this study. However, it is important to consider that the organ samples of 18 mice or 6 rats were pooled for the analysis of the binding activity in the kidneys, lungs and stomach. For the analysis of DNA binding in the liver, samples from each of the 6 rats were evaluated separately, while 3 pooled samples from 6 animals each were analysed for the mice. This makes it difficult to compare the 4 organs and the 2 species.

#### 5.5.3 Summary

Contrary to the findings of earlier studies in bacteria and yeasts, several newly cited studies found evidence of a mutagenic potential of chloroform. However, the studies in yeasts were carried out under cytotoxic conditions and without the addition of a metabolic activation system. Of the 4 studies with bacteria, 3 reported implausible results or inadequately described the study procedure. The study published by Araki (2004) essentially fulfils the current requirements of OECD Test Guideline 471. A mutagenic effect was induced by chloroform in one strain, Escherichia coli WP2/pKW101. However, this study used ethanol for the stabilization of chloroform, which may lead to the formation of the potent alkylating agents ethyl or diethyl carbonate. Chloroform yielded negative findings in another study with Escherichia coli WP2/pKW101. Gene mutations were not detected in a study with lacI-transgenic B6C3F1 mice in vivo. Therefore, the mutagenic effects in vitro were not confirmed in vivo.

The finding that DNA single strand breaks occur in rat hepatocytes only at cytotoxic concentrations (Beddowes et al. 2003) agrees with the data available to date that suggest that genotoxicity develops with the onset of cytotoxicity. In addition, lipid peroxidation was observed at the non-cytotoxic concentration of 4 mM. As described in Section 3.2, lipid peroxidation is caused by the formation of the dichloromethyl radical under reductive conditions, that is, at high concentrations and low oxygen levels. The blood:air partition coefficient for humans is 7.43 (Section 3.1), which would result in a chloroform concentration in the blood of 18 µg/l after inhalation of 2.5 mg/m^3^ (MAK value). The concentration in the blood reached after exposure at the level of the MAK value is much lower than that obtained at the concentration of 4 mM (477 mg/l) tested in vitro. Therefore, lipid peroxidation is not relevant as long as the MAK value is not exceeded. A recent in vitro study found that chloroform induces aneugenic effects. In contrast, earlier in vivo genotoxicity tests in mammals did not detect clastogenicity or aneugenicity at non-cytotoxic doses. These results were confirmed in a micronucleus test in rats carried out according to the current test guidelines.

### 5.6 Carcinogenicity

#### 5.6.1 Short-term studies

Chloroform did not have initiating effects, but there was evidence suggestive of tumour-promoting effects (Greim 2000). In a short-term study reviewed here for the first time, chloroform was given to male C57 BL/10ScSn/01a and Theiller Original mice with the drinking water. Different types of tumour cells (Ehrlich-Lettré ascites carcinoma, B16 melanoma, Lewis lung carcinoma) were then administered by intraperitoneal or subcutaneous injection or intramuscular transplantation into the flank (Capel et al. 1979).

The study design is not relevant for the evaluation; for this reason, the study is not presented in detail. Other recent studies are not available.

#### 5.6.2 Long-term studies

The carcinogenic effects of chloroform were presented in detail in the addendum from 1999. After administration by gavage, chloroform induced kidney tumours in male rats and thyroid tumours in female rats in addition to liver ­tumours in mice of both sexes. Kidney tumours were found in male rats after administration with the drinking water. Increased incidences of kidney tumours were observed also in male mice after inhalation exposure to chloroform (Greim 2000).

A carcinogenicity study investigated only the kidneys. Groups of 50 male F344 rats were exposed by inhalation for 6 hours a day, on 5 days a week, for 104 weeks. Other groups were exposed to chloroform both by inhalation and with the drinking water (see Section 5.2.1). Renal cell carcinomas were found only in the group exposed via both routes (Nagano et al. 2006). The number of adenomas and carcinomas are shown in Table 11. Further details were not provided.

**Tab.11:** Adenomas and carcinomas in the kidneys of male F344 rats (Nagano et al. 2006)

	Concentration in the drinking water [mg/l]							
	0				1000			
inhaled concentration [ml/m^3^]	0	25	50	100	0	25	50	100
estimated uptake of chloroform [mg/kg body weight and day]	0	20	39	78	45	73	93	135
renal cell adenomas	0	0	0	1	0	2	0	4
renal cell carcinomas	0	0	0	0	0	2	4	14

Two other long-term studies (Eschenbrenner and Miller 1945; Habs et al. 1983) not previously described are not suitable for inclusion in the evaluation because of their study description or design (too few animals, neutron radiation prior to administration, rudimentary description of study).

## 6 Manifesto (MAK value/classification)

The most sensitive effects are bone thickening in the nasal cavity, changes in the cells of the olfactory epithelium as well as increased cell proliferation in the liver and kidneys of mice and rats after inhalation exposure.

**MAK value. **Studies with humans investigated very high concentrations of chloroform (see Section 1). As a result, a MAK value cannot be derived from their findings.

The previous MAK value was derived from the findings of subchronic studies that established a NOAEC of 5 ml/m^3^ for increased cell proliferation in the kidneys and liver of mice and a NOAEC of 10 ml/m^3^ for the same end point in the kidneys of rats (Greim 2000; Templin et al. 1996). In 2016, the Commission began using a revised approach for the assessment of substances that cause systemic effects in inhalation studies with animals. This approach takes into account that the respiratory volume at the workplace is higher than under experimental conditions by dividing the corresponding concentrations in half. Taking into consideration that the effects may increase in severity after chronic exposure (1:2) and that the NOAEC was derived from an animal study (1:2 for extrapolation to humans), this results in a concentration of 0.63 ml/m^3^. A MAK value of 0.5 ml/m^3^ is then derived by applying the preferred value approach. This value corresponds to the previous MAK value.

In the addendum from 1999 (Greim 2000), chloroform was already reported to induce minimal effects in the olfactory epithelium of rats. Recent data for chloroform from a 2-year study with chronic inhalation confirm that the nose is one of the most sensitive target organs. Respiratory metaplasia in the olfactory epithelium as well as slight bone thickening were found at the lowest chloroform concentrations tested, 10 ml/m^3^ in rats and 5 ml/m^3^ in mice. Humans are less susceptible to the effects on the bones than rodents and the findings for metaplasia in rats are more plausible than those in mice (cf. Section 5.2.1.4 and Section 3.2.2). Furthermore, CYP2E1-mediated metabolic activity in the olfactory epithelium is much higher in rodents than in humans. Rodents are therefore assumed to be more susceptible than humans to the metabolic conversion to phosgene and thus for the induction of toxic effects in the nose by chloroform.

The effects caused by chloroform in the olfactory epithelium after exposure by inhalation are both systemic and local (see Section 2.2). For this reason, two derivation pathways are described below.

Assuming that the effects in the nose are caused by systemic absorption and distribution, a NAEC (no adverse effect concentration, equivalent to the LOAEC divided by 3) of 3.3 ml/m^3^ is estimated from the LOAEC for chloroform of 10 ml/m^3^ that was derived from a chronic inhalation study in rats. Taking into consideration the increased respiratory volume at the workplace in comparison with that of animals at rest in an inhalation study (1:2), this results in a concentration of 1.6 ml/m^3^. As humans are not more sensitive than rats, a MAK value of 1 ml/m^3^ could be derived by applying the preferred value approach.

Assuming that a local effect is induced in the olfactory epithelium (extrapolation of animal data to humans (1:2) according to Brüning et al. (2014)), a MAK value of 1 ml/m^3^, based on the NAEC of 3.3 ml/m^3^ and after applying the preferred value approach, would likewise be calculated. Overall, the systemic effects in the kidneys and liver and the effects in the nose occurred in the same concentration range. However, the olfactory epithelium of humans is assumed to be less sensitive because the metabolic activity that is necessary for the development of toxicity occurs at much higher levels in the nose of rodents. This represents an additional margin of safety between the MAK value and the level at which effects occur in the nose. Therefore, the established MAK value of 0.5 ml/m^3^ (2.5 mg/m^3^) likewise protects against effects in the nose and does not need to be changed.

**Peak limitation. **As the MAK value was derived from systemic effects, chloroform remains assigned to Peak Limitation Category II. A study (Fry et al. 1972) that investigated half-lives in test persons after oral administration found that the initial half-lives were below 1 hour, which would support an excursion factor of 1. However, there is a discrepancy in this study between the presentation of the results in the figure and the values given in the text. The NOAEC (0.625 ml/m^3^) derived from the effects in the liver and kidneys observed in animal studies is 1.25 times as high as the MAK value. With a half-life of 15 minutes, the peak concentration at an excursion factor of 2 is 1.5 times the concentration in blood after continuous exposure at the level of the MAK value (calculated based on Hartwig and MAK Commission 2017) and therefore only 20% (1.5/1.25) higher than the concentration in blood calculated on the basis of the NOAEC. As the blood:air partition coefficient for humans is much lower (about 1/3) than that of rodents (Corley et al. 1990), the body burden of humans is lower at the same external exposure level. This further justifies the assignment of an excursion factor of 2.

**Prenatal toxicity. **The following effects were observed in two prenatal developmental toxicity studies (one of which was carried out according to OECD Test Guideline 414) with whole-body inhalation exposure. Developmental delays in the form of reduced body weights and reduced crown–rump lengths were found in Wistar rats at chloroform concentrations of 30 ml/m^3^ and above. At the same time, maternal toxicity was evident in the form of reduced body weight gains and reduced feed consumption. The NOAEC for developmental toxicity was 10 ml/m^3^ (Greim 2000; Hoechst AG 1988, 1990 a, b). In Sprague Dawley rats, external malformations (shortened or missing tails and imperforate anus) and skeletal malformations (missing ribs) were observed in 3 litters at a concentration of 100 ml/m^3^. The NOAEC for these types of effects was 30 ml/m^3^ (Greim 2000; Schwetz et al. 1974). The malformations found in 3 litters at 100 ml/m^3^ are considered evidence of teratogenicity. After administration by gavage, chloroform caused developmental delays in Sprague Dawley rats and Dutch Belted rabbits concurrently with maternal toxicity. The initial effects were observed in rats at doses of 126 mg/kg body weight and day and above and in rabbits at 50 mg/kg body weight and day. The corresponding NOAELs for developmental toxicity were 50 mg/kg body weight and day (rats) and 35 mg/kg body weight and day (rabbits). Teratogenic effects were not observed (Greim 2000; Thompson et al. 1974).

After taking into account the increased respiratory volume, there is a 10-fold margin between the NOAEC of 10 ml/m^3^ for developmental toxicity in rats and the MAK value of 0.5 ml/m^3^ (2.5 mg/m^3^). The following toxicokinetic data are taken into consideration for the extrapolation of the NOAELs for development toxicity of 50 mg/kg body weight and day (rats) and 35 mg/kg body weight and day (rabbits) to a concentration in workplace air: the corresponding species-specific correction values for the rat and rabbit (1:4 and 1:2.4), the measured absorption (100%; near complete absorption in mice and rats after 48 and 96 hours (ATSDR 1997)), the body weight (70 kg) and respiratory volume (10 m3) of humans, and the assumed 100% absorption by inhalation. The concentrations calculated from this are 17.6 ml/m^3^ and 20.4 ml/m^3^ (88 mg/m^3^ and 102 mg/m^3^), respectively. These correspond to 36-fold and 41-fold margins, respectively, to the MAK value of 0.5 ml/m^3^ (2.5 mg/m^3^). As the margin between the extrapolated NOAEC/NOAEL for developmental toxicity and the MAK value is sufficiently large and a 30-fold margin was determined between the NOAEC for malformations induced in a rat strain and the MAK value, the assignment of chloroform to Pregnancy Risk Group C has been confirmed.

**Carcinogenicity. **Chloroform induces carcinogenic effects in the liver of mice, the thyroid gland of rats and the kidneys of both species. Carcinogenicity is preceded by cytotoxic effects (Greim 2000). No new carcinogenicity studies have been published since the addendum from 1999 that are relevant for the evaluation. For this reason, chloroform remains classified in Carcinogen Category 4.

**Germ cell mutagenicity. **In contrast to the earlier data, a recent study found that chloroform induces mutagenic effects in Escherichia coli WP2/pKW101. However, it could not be determined whether the solvent contributed to these effects. A study investigated the incidence of gene mutations in vivo using lacI-transgenic B6C3F1 mice (Greim 2000). Negative findings were obtained in the tests with chloroform. Therefore, the findings of the recent in vitro mutagenicity study were not confirmed in vivo. An indicator test for DNA strand breaks as well as a test for the induction of lipid peroxidation in vitro obtained positive findings only at high, mostly cytotoxic concentrations. In a study carried out according to the current test guidelines, no clastogenicity or aneugenicity was determined in a micronucleus test in vivo. Overall, the data do not suggest that chloroform is a germ cell mutagen. Therefore, chloroform has not been classified in any of the categories for germ cell mutagens.

**Absorption through the skin. **Chloroform was designated with an “H” (for substances which can be absorbed through the skin in toxicologically relevant amounts) in 1999 based on in vitro data that were supported by model calculations. The amount absorbed through the skin was calculated to be about 2000 mg and thus much higher than the amount absorbed at the MAK value (Greim 2000). Therefore, chloroform remains designated with an “H”.

**Sensitization. **There are still no findings of a sensitization potential in humans and no valid positive results from animal studies or from in vitro studies. For this reason, chloroform has not been designated with “Sh” or “Sa” (for substances which cause sensitization of the skin or airways).
